# Transparent wearable three-dimensional touch by self-generated multiscale structure

**DOI:** 10.1038/s41467-019-10736-6

**Published:** 2019-06-13

**Authors:** Kyun Kyu Kim, InHo Ha, Philip Won, Deog-Gyu Seo, Kyu-Jin Cho, Seung Hwan Ko

**Affiliations:** 1 Department of Mechanical Engineering, Seoul National University, 1 Gwanak-ro, Gwanak-gu Seoul, 08826 Korea; 2 Department of Conservative Dentistry and Dental Research Institute, School of Dentistry, Seoul National University, 28 Yeongun-dong, Chongno-Gu, Seoul, 03080 Korea; 3 Institute of Advanced Machines and Design, Seoul National University, Seoul, 08826 Korea; 4 Institute of Engineering Research, Seoul National University, Seoul, 08826 Korea

**Keywords:** Mechanical engineering, Electronic devices, Sensors and biosensors

## Abstract

Pressure-sensitive touch panels can measure pressure and location (3D) information simultaneously and provide an intuitive and natural method for expressing one’s intention with a higher level of controllability and interactivity. However, they have been generally realized by a simple combination of pressure and location sensor or a stylus-based interface, which limit their implementation in a wide spectrum of applications. Here, we report a first demonstration (to our knowledge) of a transparent and flexible 3D touch which can sense the 3D information in a single device with the assistance of functionally designed self-generated multiscale structures. The single 3D touch system is demonstrated to draw a complex three-dimensional structure by utilizing the pressure as a third coordinate. Furthermore, rigorous theoretical analysis is carried out to achieve the target pressure performances with successful 3D data acquisition in wireless and wearable conditions, which in turn, paves the way for future wearable devices.

## Abstract (web summary)

Touch technology holds potential for the development of smartphones and touchscreens, yet the conventional devices are usually built on separate pressure and location sensing units. Kim et al. show a flexible and transparent touch sensor capable of mapping the position and pressure at the same time.

## Introduction

Three-dimensional touch, also known as force touch, is a new field and becoming more widely valued in the market for its versatile function and outstanding interactions with users and accessibility to additional functionality by applying alternative touch motions. Commonly, spatial and pressure information are measured separately by simply combining two independent sensor components: a force sensor and a touch panel. Apple Inc. first released force touch technology in 2014^1^, placing force sensors underneath the four corners of the rigid glass touch panel. In addition, researchers in academia developed various transparent pressure sensors^2–7^ and integrated these sensors with commercial touch screen module^8^. However, the simply combined system has significant limitations; the sensing capability will be hindered if the force sensor is arranged at the bottom of the panel, and the transparency will be reduced in case that the force sensor is placed above the panel. Various tablet computers^9,10^ are also capable of force sensing, which indeed functions only in the presence of its own extra stylus device. As stated above, a simple combination of different sensors is usually necessary to distinguish the position and pressure signals; hence, merging two functions in a single device is very challenging and causes various practical problems although a single device 3D touch that can simultaneously sense pressure and location will be the most ideal force touch device.

Besides, developing a force sensor operating under human touch motions is one of the key goals that address these challenges. Recently reported force sensors possess geometric features which contain conductive nanomaterials to obtain the required sensing ability^11–19^. The majority of them can be classified as pyramid^6,7,20^ or dome-like structures^2,21–24^, both of which concentrate the pressure or electrons at the very edge of the structure in order to enhance the sensing capability. Typical pressures produced by normal touch are distributed in the 10–100 kPa range^25,26^, and since there is a strong relationship between the structure and the sensor performance, a concrete theoretical model that captures this relationship is necessary to describe the targeted pressure region, whereas previous studies have relied on simple analysis via curve-fitting^21,23^.

Here, we present a first demonstration of a flexible and transparent sensor which is capable of determining 3D information in a single device consisting of micro-patterned silver nanoparticle and uniformly coated metal nanowires. The unique self-generated multiscale silver micro-pattern was fabricated by a developed fast digital laser-induced thermal gradient process without the need for conventional photolithography or vacuum deposition at high temperatures, vacuum environment, or any post-processing. Self-generated wavy structures were successfully engineered through the laser-induced thermal gradient and the precise relationship between the structure and the sensing performance was examined; wide sensing capability was thereby achieved within the desired pressure regime. With the aid of the six-wire sensing mechanism proposed in this study, spatial and vertical informations can be successfully detected, as demonstrated in various applications.

## Results

### Fabrication process of 3D touch

**Fig. 1:**
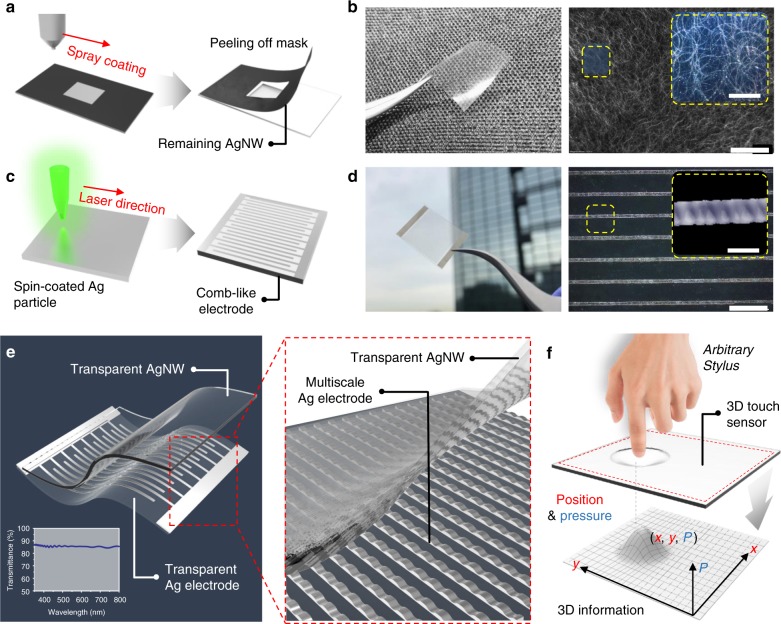
Fabrication of a wearable transparent 3D touch. **a** Schematic depicting the fabrication of the upper transparent layer. **b** Free standing AgNW–PUA composite upper layer with SEM image. The inset in the right image shows a higher magnification image displaying the ultra-thin metal nanowire network. Scale bars, 150 and 50 μm (inset). **c** Schematic depicting the fabrication of the lower transparent layer. **d** Free standing AgNP comb-like lower layer with enlarged picture of an interdigitated electrodes. Inset shows a magnified image of the surface. Scale bars, 100 and 20 μm (inset). **e** Schematic of the whole sensor system (Inset shows the transmittance of the system) with illustration of magnified image. Interdigitated lower electrodes are showing corrugated structure. **f** Illustration displaying the performance of the 3D touch

Fig. 1 illustrates the entire fabrication process and structure of the 3D touch sensor. The sensor consists of two transparent layers and the fabrication of the upper layer is shown in Fig. 1a. An ultra-thin silver nanowire (AgNW) network is uniformly sprayed onto the transfer material, covered with the desired mask pattern, and further embedded into UV curable polyurethane acrylate (PUA), the latter being designed to enhance the mechanical stability and surface flatness. Figure 1b shows a successfully fabricated free-standing transparent AgNW–PUA composite. Ultra-thin and long AgNWs were synthesized by a polyol method with extremely high aspect ratio (30 nm thick and 50 µm long). The high aspect ratio nanowires significantly enhance the surface conductivity (~20 Ω/sq) while maintaining high transparency (>95%, Supplementary Fig. 1) due to the decreased critical volume fraction of nanowires required to ensure a successive percolation network to achieve a conductive film^27–29^. The bottom layer consists of silver nanoparticles (AgNPs), which were also synthesized by a polyol method. The fabricated silver nanoparticle ink is first spin-coated on a PET substrate and then a 532 nm wavelength laser is focused at the AgNP layer to selectively convert the AgNPs into a continuous micro-sized comb-like metal pattern, as shown in Fig. 1c. The aforementioned laser process is done at low temperature and in a non-vacuum environment, which prevents significant damage of the flexible polymer from occurring during the process^30–32^. Since the sintered particles adhere strongly to the substrate, the surrounding residue could be easily removed by cleaning with polar solvents (e.g. water, ethanol (EtOH)). The transparent comb-like electrode fabricated by this process is depicted in Fig. 1d. The right image shows a microscope image of the patterned electrode with a 100 μm interval and 20 μm width. A higher magnification image is shown in the yellow-boxed inset. Both of the layers are then attached and encapsulated by PUA. The unique bi-layer sensor system is illustrated in Fig. 1e. Both the layers contribute to the excellent transparency (>85%); the transmittance (plotted on the left corner) was measured by UV–vis spectrophotometry. Macroscopically, the sensing mechanism is due to contact between the comb-like electrode array and AgNW percolation network. Microscopically, a higher external pressure forms a larger contact area between the percolation network and the self-generated corrugated structure along the electrode as demonstrated in magnified schematic, leading to more conducting pathways between the interdigitated electrodes. An overview of the sensor operation is provided in Fig. 1f. Arbitrary types of stylus, such as a finger or any type of pen can be used to operate the sensor independent of the material’s permittivity. The sensor system not only could measure the force but also recognize the contact position in simultaneous operation.

### Controlling self-generated multiscale structure

**Fig. 2:**
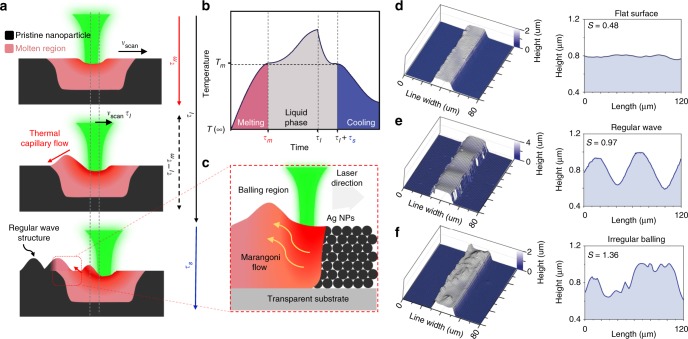
Self-generated multiscale microstructure by laser-induced Marangoni flow. **a** Time scales of microthermofluidic phenomena under optimum conditions for regular wavy structure. **b** Temporal dependence of characteristic temperature profile induced by single cycle of laser scanning. **c** Marangoni convection in molten AgNP layer induced by laser scanning. **d–f** 3D scanned image and surface profile of AgNP layer with different laser conditions with *S* = 0.48 (**d**), *S* = 0.97 (**e**), and *S* = 1.36 (**f**)

So far, non-flat surface morphology appearing in the selective laser sintering (SLS) process was considered to be a metallurgical defect, the so-called ‘balling effect’^33–35^. However, controlling the irradiation parameter with the support of sophisticated physical analysis, the morphology can be easily manipulated to a desired structure. In this way, we developed a self-generated microstructure by laser-induced spatial thermal gradient. When the laser is scanned on the spin-coated AgNP layer, a temperature difference arises between the laser spot center and the lag side. The temperature of the spot center is higher than the lag side, since the lag side has been cooled by the ambient environment. In this temperature distribution, the surface shear stress of the molten silver layer acts toward the relatively cold area, i.e., the lag side (Supplementary Fig. 2), since generally the surface tension of common liquids decreases with increasing temperature. Consequently, surface force causes Marangoni convection flow, which circulates in the right hand direction with respect to the out of plane direction (Fig. 2c). This circulating flow plays a key role in determining the surface morphology of the resultant electrode during laser irradiation (Fig. 2a). Since the surface morphology reconstruction caused by Marangoni flow occurs during the time when the AgNP layer remains in a liquid state, two time scales are major factors in laser-induced surface deformation: a time scale for Marangoni convection flow (*τ*_c_) and the characteristic time in which the AgNP layer remains liquid (*τ*_liq_). The temporal dependence of the surface temperature profile induced by laser irradiation is shown in Fig. 2b (Profiles in each four regions are discussed in Supplementary Note 1.), where *τ*_l_, *τ*_m_, and *τ*_s_ are the laser-irradiated heating time, melting time, and solidification time, respectively. The gray area denotes the time interval in which the AgNP layer remains liquid (*τ*_liq_ = *τ*_l_ + *τ*_s_−*τ*_m_). The two time scales (*τ*_s_, *τ*_m_) are negligible, since they are relatively small compared to *τ*_l_ (Supplementary Note 3); thus, the key time scales are

1 $${\mathrm{\tau }}_{{\mathrm{liq}}} \approx {\it{\tau }}_{\mathrm{l}} = \frac{L}{{{v}_{{\mathrm{scan}}}}}$$

2 $$\tau _{\mathrm{c}}\sim \frac{L}{U} = \frac{{\mu L^2}}{{|{\mathrm{{d}}}\gamma /{\mathrm{{d}}}T|{\mathrm{\Delta }}TH}},{\mathrm{\Delta }}T\sim \frac{{\dot Q}}{{kH}}$$

where *L* is the characteristic dimension (laser spot radius, 10 μm), *v*_scan_ is the laser scan speed (200 mm s^−1^), *U* is the characteristic Marangoni flow speed, *μ* is the viscosity of molten silver, |d*γ*/d*T*| is the surface tension gradient with respect to the temperature of molten silver, *H* is the height of AgNP layer, $\dot Q$ is the laser power, and *k* is the thermal conductivity of the AgNP layer (Supplementary Notes 2, 3 and 11). We quantitatively investigated the surface deformation process by introducing the Surface shaping number (*S*):

3 $${S} = \frac{{{\tau }_{\mathrm{l}}}}{{{\tau }_{\mathrm{c}}}} = \frac{{|{{\mathrm{{d}}}\gamma }/{{\mathrm{{d}}}T}|}}{{{\mu Lk}}}\frac{{{\dot Q}}}{{{v}_{{\mathrm{scan}}}}}$$

which is a dimensionless number defined as the ratio between *τ*_c_ and *τ*_l_ (≈*τ*_liq_), indicating the speed of the circulating flow compared to the solidification rate. When a large spatial thermal gradient is established in the pristine AgNP surface, surface shear stress affects the interface between liquid silver and ambient air, making it energetically unstable. A small geometrical perturbation that inherently exists on the AgNP surface causes the molten silver surface to undergo a transition to an energetically favorable state, which tends to minimize the surface free energy. Such a transition rate is inversely proportional to the mass transportation characteristic time, *τ*_c_. The process condition can be classified into three cases depending on the value of *S*. Firstly, *S* < 1 (*τ*_c_ is larger than *τ*_l_, Fig. 2d). Since the molten silver cools down rapidly, solidification occurs before the unstable liquid silver interface initiates its transition. In this instance, surface reconstruction cannot occur, and the resultant electrode remains flat. Secondly, *S*~1 (*τ*_c_ is comparable to *τ*_l_, Fig. 2e). Solidification and transition takes place simultaneously; thus, the silver is solidified and develop its structure during the intermediate morphological transition. Since the cycle of the reconstruction is analogous to the transition cycle, a regular wave structure is generated behind the laser scan direction. Lastly, *S* > 1 (*τ*_c_ is smaller than *τ*_l_, Fig. 2f). In this situation, enough time is provided to preserve the liquid phase. Since the initial geometrical perturbation is randomly distributed over the surface, this condition generates unbalanced spherical island structures, which is the lowest energy configuration of the interface. Since the laser profile and the shape are also important parameters determining the resultant surface morphology, further rigorous investigations should be required. The brief discussion regarding these parameters is found in Supplementary Note 6.

### Theoretical analysis of the sensor

**Fig. 3:**
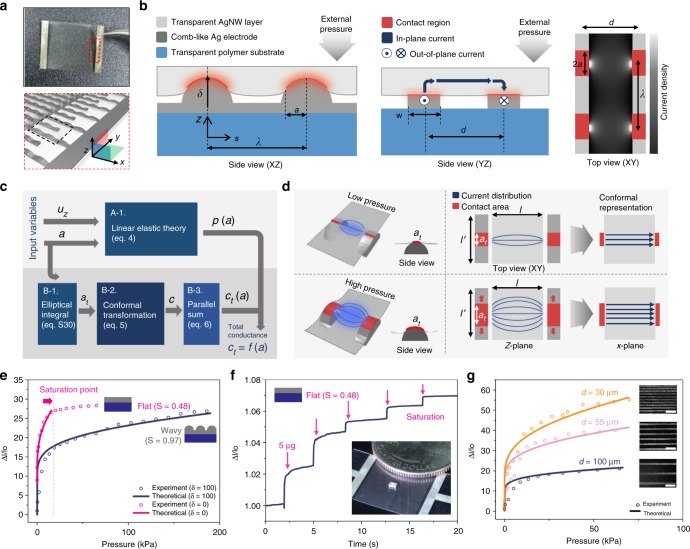
Theoretical analysis of the sensor. **a** Picture of free-standing lower comb-like electrode with magnified image in which the black dashed box denotes the control surface. **b** Illustration of electro-mechanical phenomenon with respect to the three different views in the control surface. *XZ* plane side view (left), *YZ* plane side view (center), and *XY* plane top view (right). *XY* top view containing simulation image of current distribution over the AgNW layer. **c** Diagram of the theoretical analysis. **d** Schematics of the adjusted current distribution according to different external pressure and contact area. Right side denotes the conformal representation of the equipotential line. **e** Current change for different surface morphology and comparison between the theoretical data from Eqs. (4) and (6). **f** Demonstration as a high sensitive sensor (flat morphology, *S* = 0.48). **g** Output signal distribution as a function of electrode distance compared with the theoretical data

The output signal of the sensor is mainly dictated by two factors, the pressure-dependent contact surface between the layers and the consequent change in the electrical path. A theoretical model based on contact mechanics and geometrical resistance analysis was constructed in order to predict these processes. A magnified illustration of the comb-like electrode in the red box (Fig. 3a). The geometric modeling parameters of the sensor are illustrated in the magnified images of three different views (Fig. 3b). As shown in the *XZ* plane view, the repeating unit of corrugated structure could be estimated as a single sinusoidal wave, where *δ* is the amplitude of the wave, *λ* is the wavelength, and *a* is the projected length of the contact area. Since the modulus of the lower layer (comb-like electrode on the polymer substrate, *E*_lower_ = 83 GPa) is much higher than that of the upper layer (AgNW–PUA composite, *E*_upper_ = 20 MPa), the lower layer can be considered as a rigid body; hence, we could consider that there would be a linear elastic deformation of the AgNW composite above the rigid-corrugated surface. Since the nanowire is randomly distributed over the polymer and could be treated as a continuous metal layer, the nanowire concentration would not significantly affect the output signal of the sensor (Supplementary Note 10). Therefore, the change in contact area directly affects the bridging current between the interdigitated electrode, shown in the *YZ* plane view, where *w* is the width of the electrode and *d* is the distance between the neighboring electrodes. The bridging current between the electrodes is shown in the *XY* plane view, and the current density is calculated by numerical simulation (Supplementary Note 8). Relations between the two-dimensional average pressure (*P*) and normal displacement (*u*_*z*_(*x*) = *δ* cos(2*πx*/*λ*)) can be derived by solving the two-dimensional problems of an elastic half-space (Fig. 3c(A-1), Supplementary Note 7),

4 $${{P}\left[{\mathrm{N}}\,{\mathrm{m}}^{-1}\right] = \frac{\pi^{2}{E\delta}}{\lambda (1 - \nu^{2})}\left[{\frac{1}{1!}\left( \frac{2\pi}{\lambda} \right){a}^{2}{J}_{1} - \frac{1}{3!}\left(\frac{2\pi}{\lambda} \right)^{3}{a}^{4}{J}_{3} + \frac{1}{5!}\left(\frac{2\pi}{\lambda} \right)^{5}{a}^{6}{J}_{5} - \cdots } \right]}$$

**Fig. 4:**
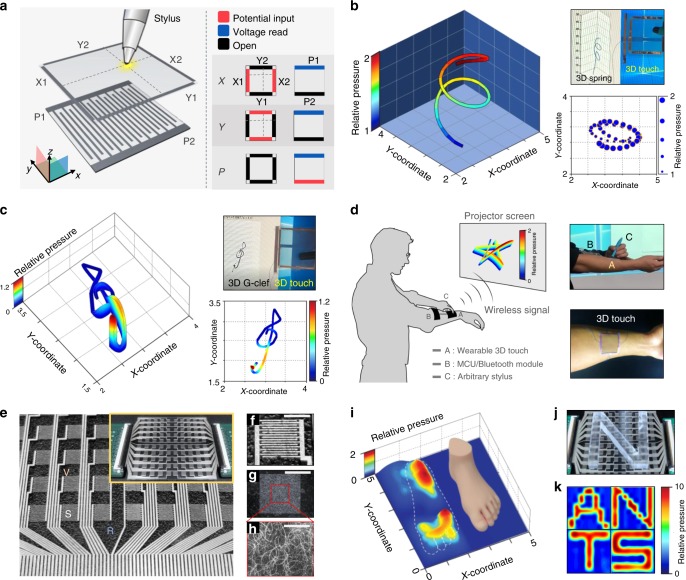
3D touch applications. **a** Working mechanism of six-wire transparent 3D touch. **b** 3D structured spring drawn by altering pen pressure: 3D touch was directly attached to a monitor while the spring is simultaneously displayed on the left. Also the pen pressure can be displayed as different line thicknesses as depicted on the right corner. **c** 3D structured G-clef drawn by 3D touch: 3D touch was directly attached to the monitor while the G-clef is simultaneously displayed on the left. The 2D figure of the G-clef is shown on the right corner. **d** Demonstration as a wearable and wireless 3D touch: Illustration of drawing 3D structured star and the real image of the attached system on forearm. **e** Picture of the 10 × 10 sensory array and the inset shows the entire system connected to the analyzer. **f** Magnified image of a single cell containing comb-like electrode. Scale bar, 3 mm. **g** The AgNW layer. Scale bar, 3 mm. **h** SEM image of the AgNW layer. Scale bar, 150 μm. **i** 3D pressure distribution of an artificial foot. Exact scale of the foot is compared next to the distribution. **j** Sample image of PDMS block forming the letter ‘N’ placed on top of the sensor array. **k** Pressure configuration showing the letters ‘A’, ‘N’, ‘T’, and ‘S’

The series *J*_*n*_ and the functional form of the actual contact area *a*_*t*_ are also given in Supplementary Note 7. As demonstrated in Fig. 3c(B-1-3), the relation between the contact length and conductance should be considered to further develop the relationship between the external pressure and output current signal of the sensor. As shown in Fig. 3d, a higher pressure generates more conducting pathways, which causes an increase in the output current at a fixed supply voltage. However, since the equipotential line is non-linear, the conductance cannot be easily calculated by the simple electric conductance relation*, G* = *σA*/*l*, where *σ* is the conductivity, *A* is the cross-sectional area, and *l* is the length of the conductor. In this situation, the conductivity could be calculated by mapping every point of the physical plane (*Z*-plane) conformally to a corresponding auxiliary plane (*χ*-plane), the so-called, conformal representation. Among such methods, we used the Schwarz–Cristoffel transformation (Fig. 3c(B-2)). As shown in Fig. 3d, by transforming the extremities of the contact area on the *Z*-plane, the equipotential line becomes linear on the *χ*-plane. Consequently, the geometrical resistance (*G*) can be approximated as

5 $${G} \cong \frac{{{K}\prime \left( {k} \right)}}{{{K}\left( {k} \right)}},{k} \cong {\mathrm{tanh}}\left[ {\left( {\frac{{\pi }}{2}\left( {\frac{{Y}}{{X}}} \right)} \right.} \right]$$

where *X* = *l/l*′, *Y* = *a*_*t*_/*l*′, and *K* denotes a complete elliptic integral of the first kind. A detailed derivation and an approximation of single cell conductance can be found in Supplementary Note 9. Since the entire sensor system is a combination of these microcells, the total conductance of the sensor can be calculated by a parallel sum approximation (Supplementary Note 8, Fig. 3c(B-3)). Therefore, the total conductance is given by

6 $${C}_{{\mathrm{{t}}}} = {N\sigma t}\frac{1}{{G}}\sim O(10^{ - 1})\frac{1}{{G}}$$

where *N* is the number of corrugated microcells O(10^2^), *σ* is the conductivity of AgNW network O(10^4^) S/m and *t* is the thickness of the AgNW layer O(10^–7^) m. Thus, the correction factor is on the order of 10^−1^ in SI units. Taking the correction value of 5.6^−1^ and combining Eqs. (4)and (6), we could finally deduce the theoretical model of the sensor which explains the relation between the pressure and output current signal. Theoretical values were found to match perfectly with the experimental values, as depicted in Fig. 3e, g. To further investigate the performance of the sensor, we controlled two parameters, *δ* and *d*, which are amplitude and distance, respectively (Fig. 3e). An electrode with a flat surface (*δ* = 0) operates as a contact between metal plates, i.e., the contact will form instantly. The pressure sensitivity seems to be extremely high; however, the sensing range is relatively small (<10 kPa). As a demonstration, a flat surface sensor is capable of detecting consecutive loading of five microcapacitor chips (5 μg each), where the inset shows the sensor and the microcapacitor chip compared with a US quarter coin (Fig. 3f). An electrode with a corrugated surface (*δ* = 100 μm), has an enhanced sensing range, attributed to the multiscale structure (Fig. 3e), which makes it possible to detect inputs in the high-pressure regime (10–100 kPa) produced by daily life^25,26^, and the applications of this structure will be covered in Fig. 4. The distance between the electrodes also affects the sensor performance, as shown in Fig. 3g. A narrower electrode approaches the metal plate, and due to the same phenomenon above, increased sensor sensitivity can be observed in the figure. Furthermore, the sensor endures 30,000 pressure cycles with 24 ms response time (Supplementary Note 13). These results successfully demonstrate the accurate performance of the sensor, and from the theoretical analysis, the performance of the sensor is freely adjustable and can be implemented in various applications for different purpose.

### 3D touch applications

The working mechanism of the transparent 3D touch is illustrated in Fig. 4a. The sensor consists of six independent wires, four at the upper AgNW layer and two at the bottom interdigitated electrode. Wires are colored depending on their electrical status: potential input, voltage read, and open state. In order to detect the 3D signal, three steps of voltage switching are necessary. First, an equipotential distribution parallel to the *x*-axis is generated through the AgNW layer (*X*1–*X*2). The touched *x*-coordinate will be measured by the bottom read line (P1), detecting the voltage at the contact point. The bottom electrode is fabricated in the *S*~1 condition, where the surface is regularly corrugated, which enables it to detect a wide range of pressures. In the same manner as the *x*-direction, the *y*-coordinate will be detected by applying a potential difference through the *y*-axis (*Y*1−*Y*2). After the coordinate detection, the electrode at the upper layer is switched as an open state, preventing current leakage through the upper electrode. Voltage is then applied in the remaining bottom electrode (P2), detecting the increased conducting pathways between the comb-like electrodes. The detailed working mechanism is illustrated in Supplementary Note 14. As shown in Fig. 4b, the 3D touch was directly attached to the monitor screen and successfully able to draw a 3D-structured object. We drew a continuous circle with increasing pressure, and a 3D-structured spring was simultaneously generated on the screen (Supplementary Movie 1). Furthermore, drawing a 3D-structured G-clef was accomplished as depicted in Fig. 4c (Supplementary Movie 2). An upper view of the G-clef is shown on the right corner, where the high-pressure region can be observed on the tail of the G-clef. As a proof-of-concept demonstration of its application as wireless wearable 3D touch, we combined the sensor with an integrated circuit board, MCU, and Bluetooth module as illustrated in Fig. 4d. The 3D touch was conformably attached to the forearm, and successfully transmitted the 3D information of a hand drawn star (Supplementary Movie 3). To demonstrate the device’s expandability, a sensor was fabricated with a sensing network of 100 pixels (10 × 10) consisting of 5 mm × 5 mm-sized cells (Fig. 4e, V, R and S denote the voltage line, read line, and sensor, respectively). A magnified image of the sensor and the nanowire array is shown in Fig. 4f–h. The sensor is capable of detecting a miniaturized PDMS foot (Fig. 4i), exhibiting an excellent pressure distribution (Supplementary Fig. 12). Also, four different pieces of PDMS forming the letters “ANTS” were placed on top of the sensor (Fig. 4j), showing a perfect pressure configuration, as shown in Fig. 4k.

## Discussion

In summary, we have created a new type of transparent 3D touch, for the first time (to our knowledge), which operates in a single device. The integrated sensor was fabricated through mask-less laser processing of Ag nanoparticles and spray coating of Ag nanowires. The conditions for the various multi-scale structures generated by laser thermal gradient were evaluated and characterized by a dimensionless surface shape number, *S*. The mechanism of the sensor was precisely investigated by contact mechanics and conformal mapping of the current distribution and a concrete correlation between the surface morphology and the sensor performance was found. The analytical model relating them laid the foundation for determining the design and patterning parameters of the sensor for various applications. With the assistance of the newly suggested six-wired system, the sensor could assign 3D sensing capability to various surfaces while remaining nearly imperceptible to the user. This 3D touch also demonstrated perfect operation in a wearable and wireless environment. This system can have a great impact in the implementation of future wearable devices and brings a powerful new dimension to human–machine interactions.

## Methods

### Synthesis of ultra thin and long AgNWs

Synthesis of the AgNWs in this research uses a modified polyol process and a one-pot process, where all reagents are dumped into a tri-angular flask at once to prevent the thermal chemical reaction. Briefly, a 0.4 g of PVP (*M*_w_~360,000) and a 0.5 g of silver nitrate (AgNO_3_) are sequentially dissolved in a 50 mL of ethylene glycol (EG) using a magnetic stirrer. The stirrer is carefully removed from the mixture solution once all chemicals are thoroughly dissolved. Then, 800 μl of both CuCl_2_·2H_2_O (3.3 mM in EG) and CuBr_2_ (1.68 mM in EG), are rapidly injected into the mixture and stirred mildly. Then, the mixture solution is suspended in a preheated silicone oil bath at 150 °C. The growth reaction of AgNWs is maintained at the elevated temperature for an hour. When the growth is finished, the resultant solution is separated using acetone first and cleaned by repeating dispersing in EtOH and centrifuging at 4000 rpm for 10 min. This repetitive cleaning process is done at least three to four times to securely remove the organic residues. The purified AgNW solution is re-dispersed in EtOH with 0.1 mg ml^−1^ concentration.

### Synthesis of silver nanoparticle ink

Modified polyol method was used to synthesize AgNPs. 0.25 mol/l of silver nitrate was dissolved in EG with 0.02 mol/l of PVP (*M*_w_~10,000). The reaction condition (150 °C and 150 rpm magnetic stirring) was maintained until the synthesis was completed. The synthesized AgNPs were cleaned with acetone and EtOH three times and collected by centrifugation of 7000 rpm for 30 min. The purified AgNPs were re-dispersed in EtOH for the use.

### Preparation of AgNW layer by spray coating

AgNW layer was prepared by large area spray-coating method. The spray coater (automatic spray coater, Hantech) deposits AgNW solution on masked flexible substrate with conditions height 25 cm, back pressure 0 kPa, speed 20 mm/s, and spraying pressure 60 MPa (Supplementary Fig. 1a). The arbitrary flexible substrate should be pre-heated to 60 °C which evaporates the polar solvent of AgNW solution. Fabricated AgNW layer has outstanding uniformity with high trasmittance (Supplementary Fig. 1b).

### Preparation of AgNP electrode by direct laser writing

Synthesized AgNP ink (20 wt%) is spin coated on flexible substrate with 200 rpm and 1 min condition, which allows fine deopsition and evaporation of the solvent. The optical system consisted of continuous wave 532 nm laser (Sprout-G-5W, Lighthouse Photonics, continuous-wave, beam profile: Gaussian, beam ellipticity: <1.0:1.1, beam quality: 1.0–1.1, Supplementary Fig. 15), galvano-mirror (hurrySCAN II, Scanlab), and *f*-theta telecentric lens at *f* = 103 mm is used to SLS and patterning. The rasidual in patterened sample is washed by using polar solvents such as DI water. Detailed information of laser sintered electrodes can be found in Supplementary Note 12.

### Measuring the response of the sensor

A home-made system containing high-resolution linear actuator and load cell (Futek) was prepared. The system can provide an external pressure up to 200 kPa and the electrical signal was simultaneously recorded by digital multimeters (Keithley 2002, Keithley).

### Measurement of surface profile of the AgNP layer

The surface morphology of fabricated AgNP electrode had been investigated by using non-contact 3D surface profiler (NANO View-E1000, NANO system). Selected samples under various *S* numbers are profiled in ×50 lens mode, and clearly shown their 3D surface morphology.

## Supplementary information

Supplementary Information

Description of Additional Supplementary Files

Supplementary Movie 1

Supplementary Movie 2

Supplementary Movie 3

## Data Availability

The data that support the findings of this study are available from the corresponding authors upon reasonable request.
