# Supplementary material for: Transparent wearable three-dimensional touch by self-generated multiscale structure
Source: Nat Commun. 2019 Jun 13;10:2582. doi: 10.1038/s41467-019-10736-6 (PMC6565712; doi:10.1038/s41467-019-10736-6)
Supplement: Supplementary file 1 — Supplementary Information [file 41467_2019_10736_MOESM1_ESM.pdf]

Supplementary Information

Transparent wearable three-dimensional touch by self-generated multiscale structure

Kim et al.

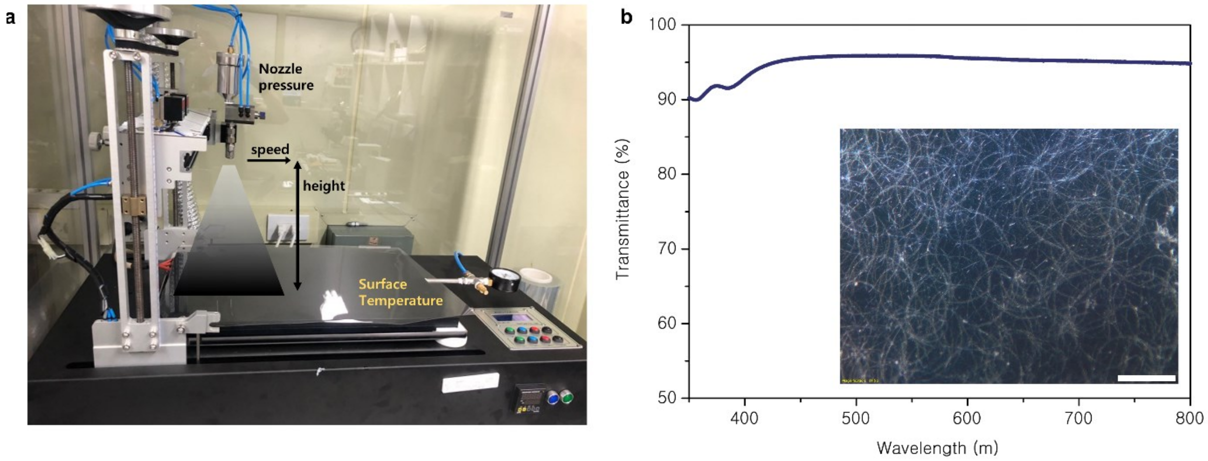

Supplementary Figure 1 | Flexible and transparent conducting layer: Spray coated AgNW film. **a.** Automatic spray coating system. Main control variables are shown in the figure. **b.** Transmittance of fabricated AgNW conducting film. Scale bar, 50 µm.

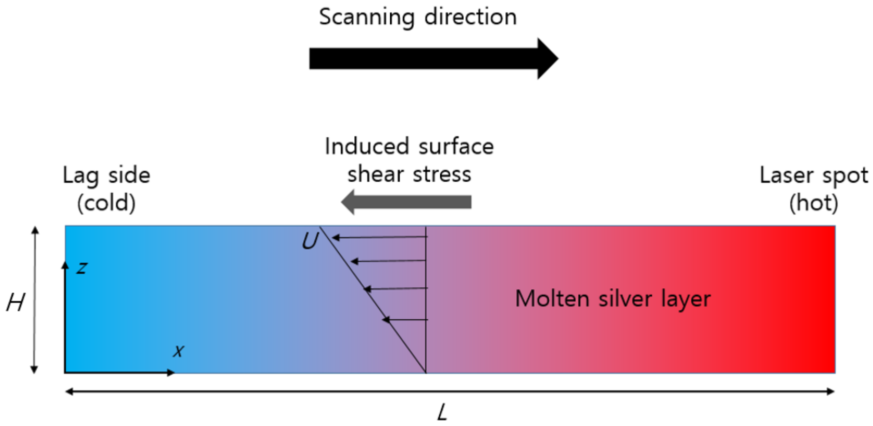

Supplementary Figure 2 | Schematic diagram of laser heated unit cell. The characteristic length of scanning direction, L is chosen to be half width of laser intensity, 10 µm. Layer height, H is 1 µm. The lubrication approximation can be applied; inner flow velocity profile is assumed linear.

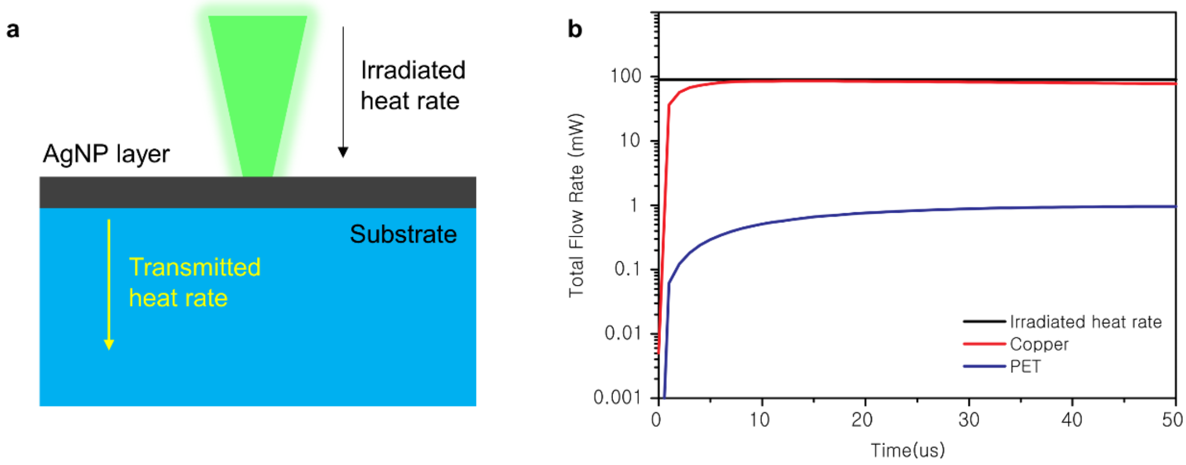

Supplementary Figure 3 | Simulation for verifying the adiabatic boundary condition. **a.** Irradiated heat rate is absorbed to AgNP layer, remained heat transmitted to substrate. **b.** The heat flows to the substrate in different ways depending on thermal diffusivity condition

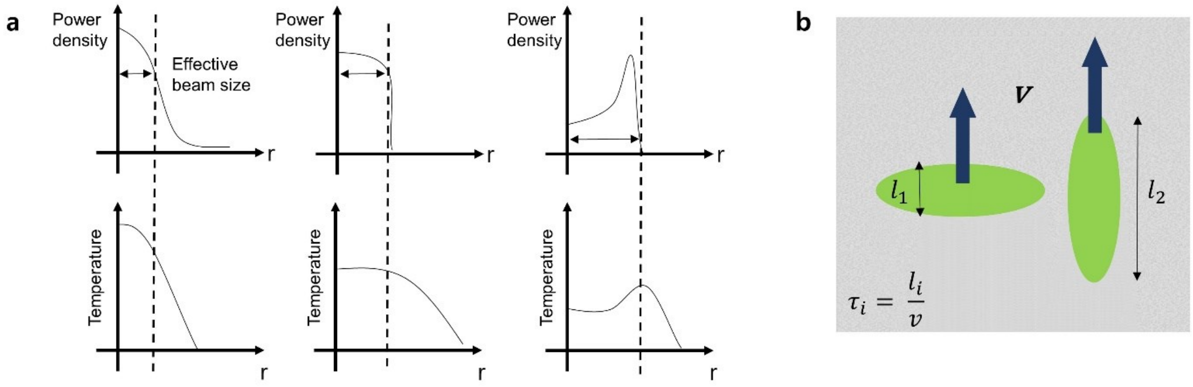

Supplementary Figure 4 | Classification of laser profile and shape. **a.** 3 cases of smooth laser beam profiles with induced temperature field expectations. **b.** laser times following the elliptical beam shape.

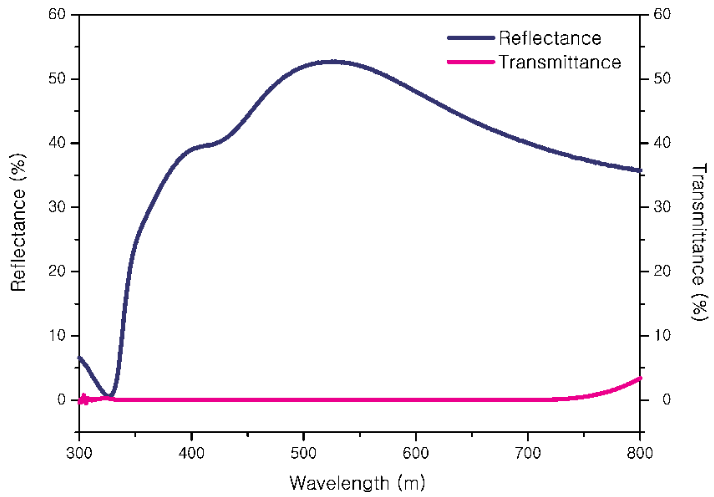

Supplementary Figure 5 | Optical property of lower layer. Absorbance at 532nm wavelength was calculated using reflectance and transmittance.

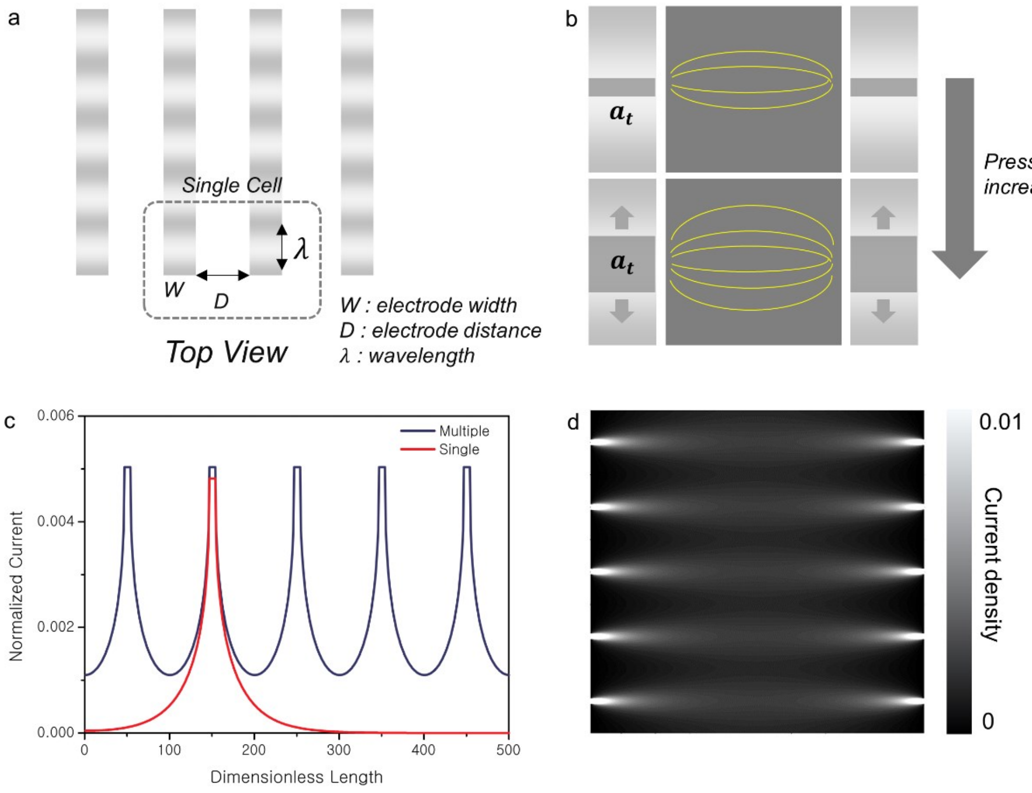

Supplementary Figure 6 | Simulation of the parallel micro resistance. **a.** Repeated unit cell of interdigitated electrode. Periodical distance is its wavelength λ. **b.** Nonlinear stream line of electrical current of each single cell. As applied pressure increases, contact area a_t is also magnified resulting larger conductance. **c.** Vertical current density averaged along horizontal direction. The current density of single micro resistor enveloped by current density of multiple network. Normalized resistance of single cell is 2.38 while multiple's is 0.498. **d.** 2D view (same as supplementary Figure 2b) of current density of multiple network. Each cell's current distribution is well isolated.

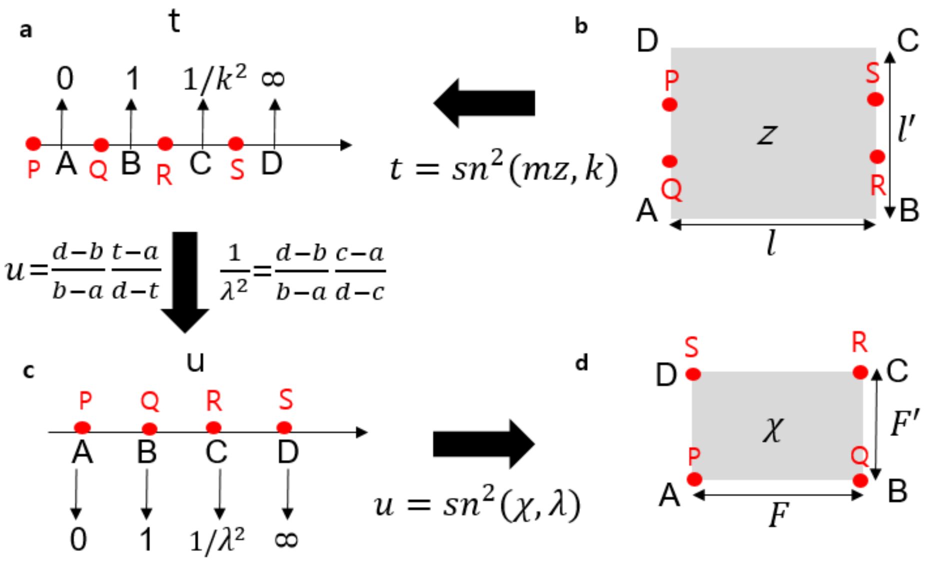

Supplementary Figure 7 | Three steps of conformal transformation, from Z-plane to χ-plane. **a.** t plane, each vertex is arranged along real axes, horizontal axes. **b.** Z-plane, geometrical representation of single micro resistor on imaginary plane. **c.** u-plane, vertexes of micro resistor and electrodes are matched together by t-u transformation. **d.** χ-plane, newly positioned vertexes of electrodes are merged to each four corners. The geometrical resistance can be calculated by using F, F'.

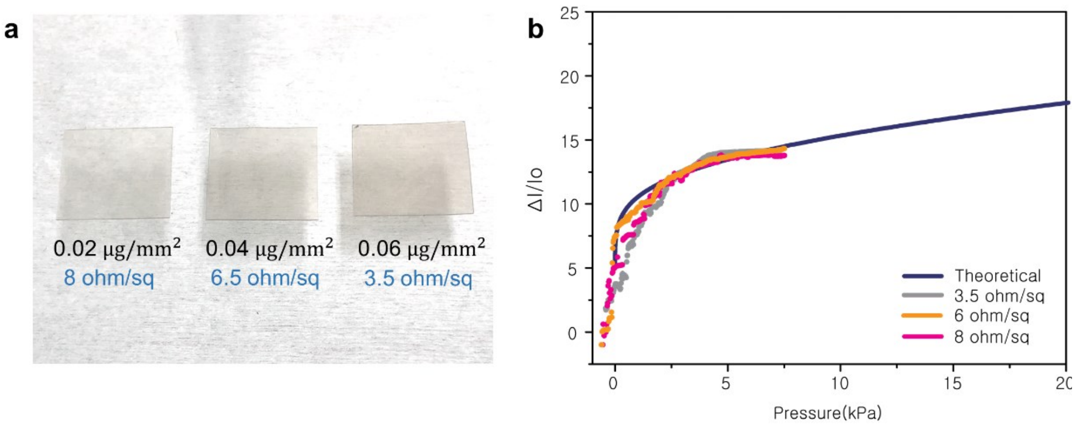

Supplementary Figure 8 | Output signal change with altering nanowire density **a.** Prepared nanowire composite with different density. **b.** Output signal with altering nanowire density.

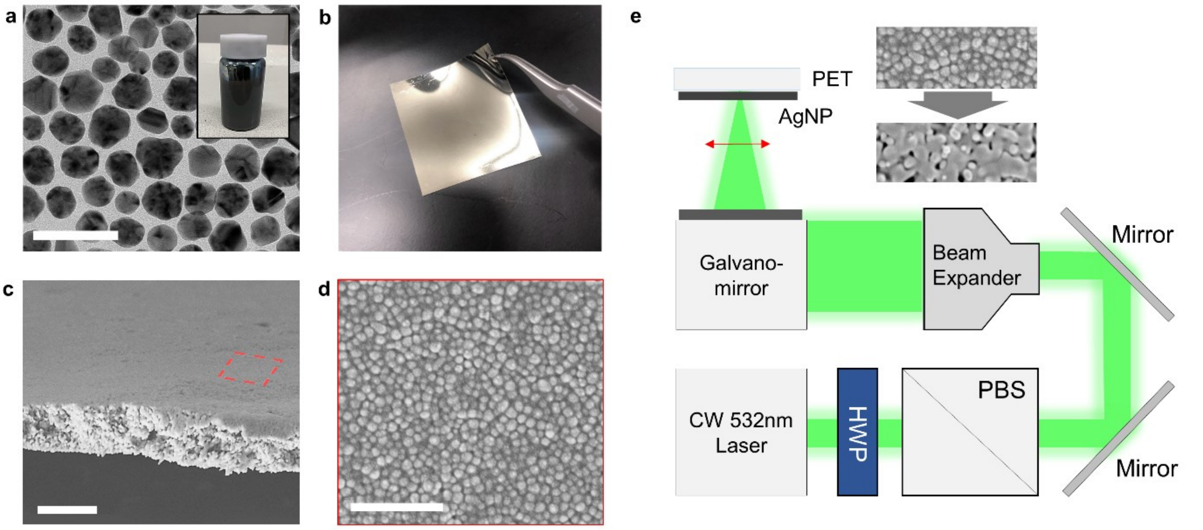

Supplementary Figure 9 | Basic information for laser sintering process. **a.** TEM image of Ag nanoparticle. Scale bar 100 nm. **b.** Picture of the spin-coated Ag nanoparticle layer on PET substrate. **c.** SEM image of the spin-coated layer. Scale bar 1 μm. **d.** Magnified SEM image of deposited particle layer. Scale bar 400 nm. **e.** Optical setting of the laser sintering system, where inset represents the melted particle after laser irradiation.

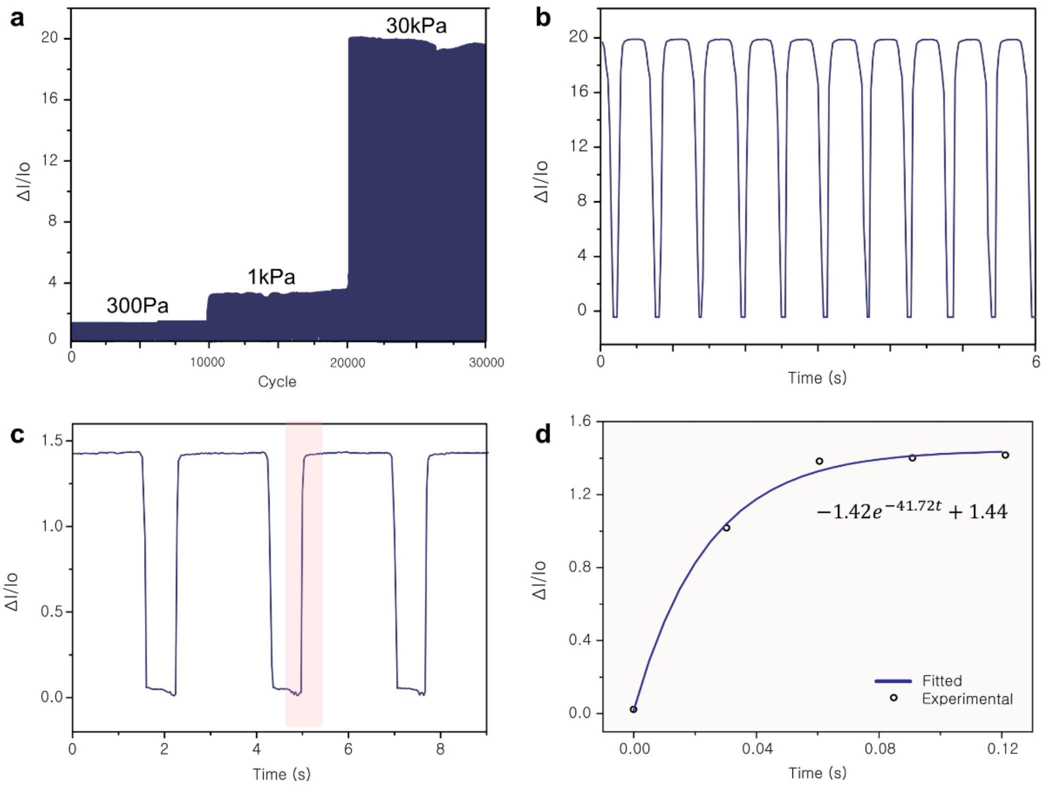

Supplementary Figure 10 | Dynamic durability and response time of the sensor. **a.** Cyclic test in different pressure. **b.** Output response in 30 kPa pressure loading. **c.** Response cycle. **d.** Exponential curve fitting and time constant calculation.

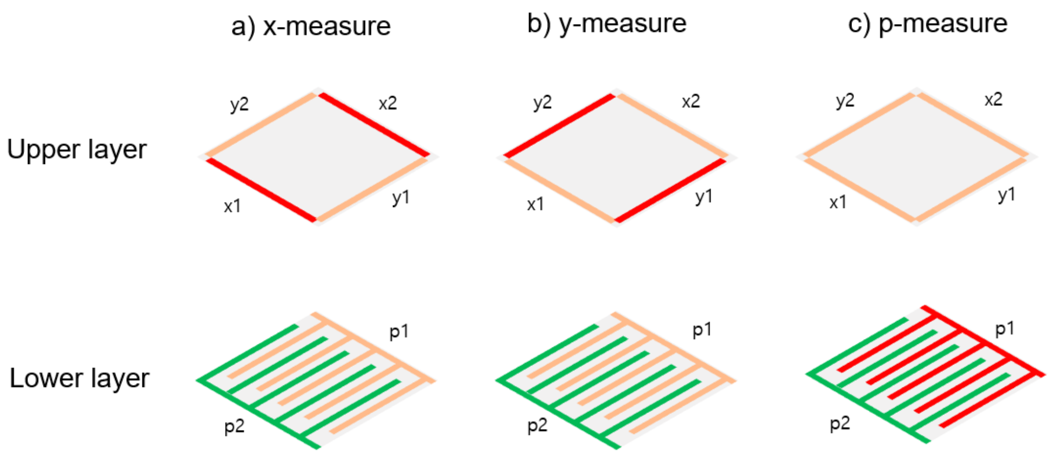

Supplementary Figure 11 | Operation of 6-wire measuring system. **a, b.** red pins represent electrical potential input electrodes, green one is voltage reading pin. Other yellow pins are in electrically open state. In 2D data measuring procedure, potential difference is applied between two facing red pins. The measuring part, green pin read the voltage of contacted point, position information. **c.** The pressure measuring mode is working in condition that yellow pins are all in open and red pin is in high. Reading pin measures the voltage between reference resistor and the green pin, which represent the resistance of pressure sensor.

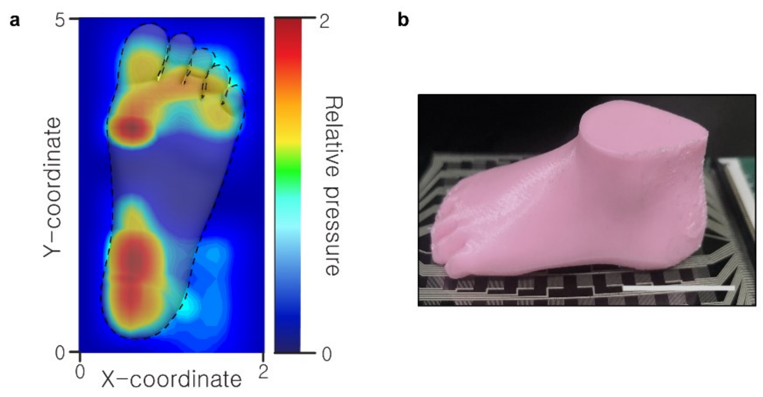

Supplementary Figure 12 | 3D pressure distribution of an artificial foot. **a.** 2D pressure distribution of the artificial foot **b.** Artificial PDMS foot placed on top of the sensor array.

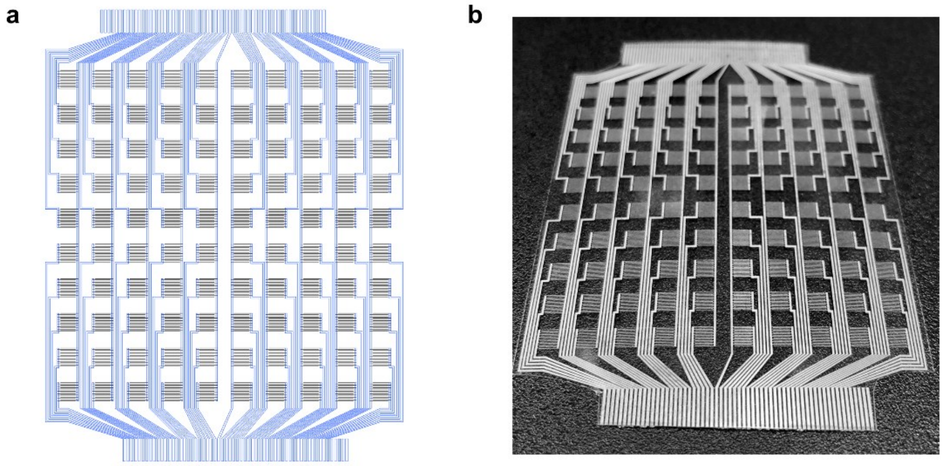

Supplementary Figure 13 | 10×10 pressure sensor pixel. **a.** CAD layout of 10×10 pressure sensor pixel. **b.** Fabricated integrated sensors and data lines. Line and sensors are all single processed, laser direct writing. Data pins are connected to FPC connector.

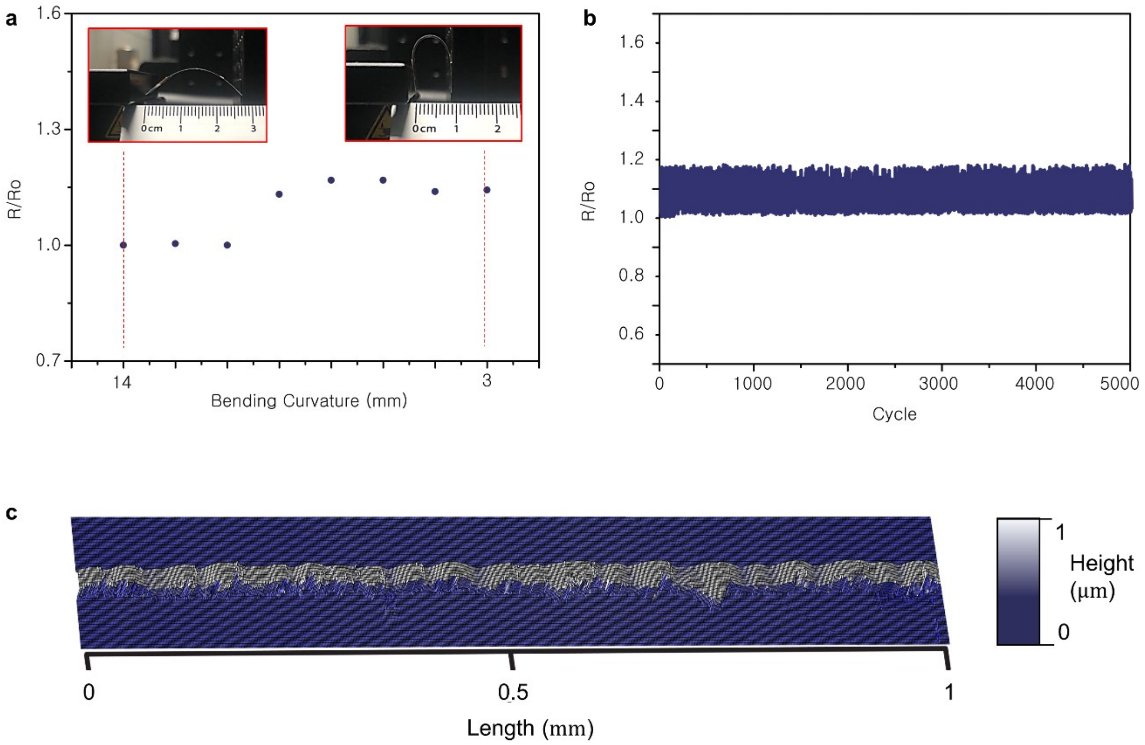

Supplementary Figure 14 | Flexibility and uniformity of the Ag electrode. **a.** Electrical stability under bending. **b.** Cyclic response under 3 mm bending curvature **c.** Uniformity of the corrugated structure

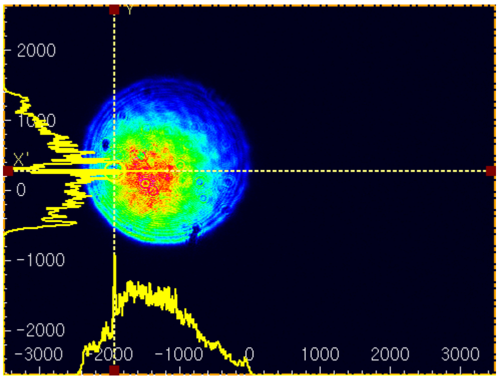

Supplementary Figure 15 | Laser profile measurement. The profile of the laser is detected from the beam analyzer (CCD Camera Beam Profiler, BC106N-VIS, Thorlabs)

Supplementary Note 1. Time scales regarding the temperature induced by laser irradiation

To irrustrate a thermal phenomenum during laser sintering, 'Lumped capacitance model' which can predict the temperature of solids experiencing a sudden change in its thermal environment[1] was employed. The model stands on the assumption which describes gradient of temperature field in a solid as zero, that is, thermal conduction in a solid would be negligible due to the fast changing of thermal environment. A situation of heat transfer for high conductive material can be also illustrated by such a way. Since the AgNP layer heated by laser irradiation also experiences extremely fast heat exchange, 'Lumped capacitance model' could explain the temporal change of representative temperature of the system. Basic derivation of the model starts from energy balance of control volume,

$$\rho c_{\mathrm{p}} V \frac{dT}{dt} = -hA(T - T_{\infty}) + \dot{Q} \tag{1}$$

where $\rho c_{\mathrm{p}} V$ is heat capacity of control volume, T is characteristic temperature, h is convective heat transfer coefficient, A is the area of heat exchange, T_{∞} is ambient temperature, and $\dot{Q}$ is volumetric heat input. With proper initial condition (say $T(0) = T_{\infty}$) for heating section (regime (i), (ii) in Figure 2b), above first order ordinary differential equation gives us the solution form of exponentials,

$$T(t) = \frac{\dot{Q}}{\rho c_{\mathrm{p}} V}\left[1 - \exp\left(-\frac{hA}{\rho c_{\mathrm{p}} V} t\right)\right] + T_{\infty} \tag{2}$$

The solution for cooling part (regime (iii), (iv) in Figure 2b) can be obtained imposing an initial condition as $T(0) = T_{\mathrm{i}}$ with $\dot{Q} = 0$

$$T(t) = (T_{\mathrm{i}} - T_{\infty}) \exp\left(-\frac{hA}{\rho c_{\mathrm{p}} V} t\right) + T_{\infty} \tag{3}$$

In consequence, the general temporal evolution of representative temperature of the system is expressed by exponentials having time constant related to heat capacity. In a one cycle of laser scanning, there are distinctive steps expected during evolution of temperature. Firstly, a solid AgNP layer is heated up until its melting point (regime (i)). The time required to melt a solid silver layer is defined as τ_{m}. Secondly, silver layer is heated until laser irradiation terminated (regime (i), (ii)). Laser time which heat input $\dot{Q}$

exists is indicated as τ_1. Note that each time constants of exponentials are different since heat capacity of solid silver is quite deviated from liquid silver's, and between two regions, constant temperature section must be inserted because of melting transition. After heating section (regime (i), (ii)), natural cooling (regime (iii), (iv)) would be occurred, time constants for each phase are same as heating section, but this statement is not inferring that rate of change of temperature is also same, since heating and cooling situation have different multiplied coefficient of exponential.

Supplementary Note 2. Marangoni convection flow characteristic time scale

When thermal gradient is induced by laser along scanning direction, circulating flow toward lag side would be driven by generated surface shear stress. We considered driving and drag forces acting on interface for determining the characteristic velocity of the flow in film layer (Supplementary Figure 2). According to the lubrication theory, inertia terms in the Navier-Stokes equation could be negligibly small under condition that system we are interested has very small height compare to system characteristic length, *i.e.* film flow. In these circumstances, the force balance of differential surface can be read as follow.

$$\overleftrightarrow{\tau} \cdot d\mathrm{A} = \boldsymbol{\nabla}(\gamma) dA \tag{4}$$

where dA is infinitesimal area of heated region. Left hand side is viscous shear force where $\overleftrightarrow{\tau}$ is surface stress tensor and right hand side is driving force where $\boldsymbol{\nabla}(\gamma)$ gradient vector of surface tension. Since laser beam has radial symmetry, surface tension gradient acts effectively to *x* direction on *z* surface. Therefore, except τ_{xz} all other stress tensor component would be negligible. The force balance relation can be reduced considering only radial component (x direction),

$$\tau_{\mathrm{xz}} dA = \frac{d\gamma}{dx} dA \tag{5}$$

$$\mu \frac{du_{\mathrm{x}}}{dz} = \frac{d\gamma}{dx} = \frac{d\gamma}{dT}\frac{dT}{dx} \tag{6}$$

Applying the order-of-magnitude analysis to Supplementary equation 6,

$$\mu \frac{U}{H} \sim \left| \frac{d\gamma}{dT} \right| \frac{\Delta T}{L} \tag{7}$$

where μ is viscosity of liquid silver, U is characteristic velocity of Marangoni convection, γ is surface tension of liquid silver, and ΔT is characteristic temperature difference. Thus, the scaling of characteristic velocity of Marangoni convection becomes,

$$U \sim \frac{H\Delta T}{\mu L} \left| \frac{d\gamma}{dT} \right| \tag{8}$$

Finally, the scaling of characteristic time of Marangoni convection is that

$$\tau_{\mathrm{c}} \sim \frac{L}{U} = \frac{\mu L^2}{H\Delta T}\frac{1}{\left| {}^{d\gamma}/_{dT} \right|} \tag{9}$$

ΔT will be discussed in detail with heat transfer analysis, Supplementary Note 3.

Supplementary Note 3. Heat transfer consideration & thermal time scale

The order of temperature difference ΔT between the center of laser spot and lag side can be scaled by comparing magnitudes of each terms in integrated heat conduction equation.

$$\rho c_{\mathrm{p}} \frac{\partial T}{\partial t} = k\nabla^2 T + \dot{q} \tag{10}$$

where ρc_{p} is volumetric heat capacity of layer, k is thermal conductivity. Using geometrical and thermal remarks of molten silver layer system (Supplementary Figure 2, Note 5), integrated both side with respect to *x, z* direction,

$$\rho c_{\mathrm{p}} H \frac{\partial}{\partial t} \int_0^L T dx = \ kH \int_0^L \frac{\partial^2 T}{\partial x^2} dx + q'' L \tag{11}$$

$$\rho c_{\mathrm{p}} HL \frac{\partial}{\partial t} \Delta T = \ kH \left(\left.\frac{\partial T}{\partial x}\right|_{r=L} - \left.\frac{\partial T}{\partial x}\right|_{r=0} \right) + q'' L \tag{12}$$

The order of magnitude analysis transforms Supplementary equation 12 to

$$\rho c_{\mathrm{p}} HL \frac{\Delta T}{\tau_{\mathrm{l}}} \sim \ kH \frac{\Delta T}{L} + q'' L \tag{13}$$

$$\Delta \mathrm{T} \ \sim \frac{q'' L^2}{\frac{\rho c_{\mathrm{p}} H L^2}{\tau_{\mathrm{l}}} - kH} = \ \frac{q'' L^2}{(\rho c_{\mathrm{p}} v_{\mathrm{scan}} L - k) H} \tag{14}$$

$q'' = a q''_{\mathrm{laser}}$ where a is absorbance of silver at 532nm and q''_{laser} is irradiated laser heat flux. But temporal contribution term has one dimensional parameter that we treat as micro scale. For vivid picture of such a dimensional property of laser induced temperature field, we had investigated comparing laser scan speed and thermal conduction diffusion speed. With proper condition of laser micro process, we can expect thermal diffusion speed be much faster than scanning speed for concrete formation of electrode. Thus,

$$v_{\mathrm{conduction}} \ \sim \frac{\alpha}{L} = \ \frac{k}{\rho c_{\mathrm{p}} L} \tag{2}$$

$$\frac{v_{\mathrm{scan}}}{v_{\mathrm{conduction}}} \sim \frac{v_{\mathrm{scan}}\rho c_{\mathrm{p}}L}{k} \ll 1 \tag{3}$$

$$v_{\mathrm{scan}}\rho c_{\mathrm{p}}L \ll k \tag{4}$$

Under condition usually implemented in laser experiment, we could have checked above assumption, left hand side of inequality is $9.63\ \mathrm{mW\ m^{-1}K^{-1}}$ while the right hand side is $180\ \mathrm{W\ m^{-1}K^{-1}}$ which has 4 order of magnitude larger than left side (Supplementary Table 3). With this comparison, the characteristic temperature difference is expressed as:

$$\Delta T \sim \frac{q''L^2}{kH} = \frac{\dot{q}}{kH} \tag{5}$$

Time scales related temporal change of temperature, $\tau_{\mathrm{m}}, \tau_{\mathrm{l}}$ and τ_{s} are compared for understanding major factor of the process. Each times are scaled by ratio between heat exchange rate and related heat amount except τ_{l} which can be scaled explicitly.

$$\tau_{\mathrm{m}} \sim \frac{\rho H L^2\{\varphi_{\mathrm{m}} + c_{\mathrm{p}}(T_{\mathrm{m}} - T_{\infty})\}}{\dot{q}} = \frac{\rho H\{\varphi_{\mathrm{m}} + c_{\mathrm{p}}(T_{\mathrm{m}} - T_{\infty})\}}{q''} \sim H \tag{6}$$

$$\tau_{\mathrm{l}} \sim \frac{L}{v_{\mathrm{scan}}} \sim L \tag{20}$$

$$\tau_{\mathrm{s}} \sim \frac{\rho c_{\mathrm{p}} H L^2 \Delta T}{h L^2 \Delta T} = \frac{\rho c_{\mathrm{p}} H}{h} \sim H \tag{21}$$

where φ_{m} is latent heat of silver for melting, T_{m} is the melting temperature of silver and h is convective heat transfer coefficient of AgNP layer. These relations show that τ_{l} is the largest time scale since $H < L \ll 1\ \mathrm{m}$, while other factors are intensive thermodynamic property except q''. Furthermore, other two time scale have large denominator compare to τ_{l}. Input laser heat flux q'' is extremely large from nature of laser irradiation, effective natural convective heat transfer coefficient, h is enhanced with large surface area. Meanwhile, calculation of time scales[2] verifies above observation.

$$\tau_{\mathrm{m}} = \frac{1}{\alpha}\left\{\frac{(T_{\mathrm{m}} - T_{\infty})k}{I_{\mathrm{a}}}\right\}^2 \text{, for surface absorption} \tag{22}$$

Irradiated optical energy cannot propagate further the absorption length, the inverse of absorption coefficient for irradiated laser beam wavelength. When absorption length is very much shorter than

thermal length of material, the optical energy converts to heat at the only thin layer whose height would be order of absorption length. The optical length of Ag for 532nm electromagnetic irradiation[3] is 12.3nm while thermal length is 13μm which has 3 order of magnitude larger than optical length.

Solidification time is expressed as,

$$\tau_{\mathrm{s}} = \frac{H^2}{4\zeta^2\alpha}(1 + \frac{2\zeta\sqrt{\alpha\tau_{\mathrm{l}}}}{H}) \tag{7}$$

Where ζ is the diemsionless constant, physically reasonable values within closed interval [0.25,1].

We calculated above three time scales[2] with following conditions: $v_{\mathrm{scan}} = 200\ \mathrm{mm\ s^{-1}}$ and $\dot{q} = 90\ \mathrm{mW}$. As expected, τ_{l} is dominant time scale in heating & solidification process (Supplementary Table 1), we concluded that the liquid time of AgNP layer is approximately equal to τ_{l}.

Supplementary Note 4. Surface shaping number (S)

As mentioned in manuscript, the surface morphology of AgNP layer is governed by the non-dimensional number S, ratio between Marangoni convection characteristic time τ_{c} and liquid time τ_{liq}. Following the logical step in Supplementary Note 1, 2, we determined the number S as below.

$$\mathrm{S} = \frac{\tau_{\mathrm{liq}}}{\tau_{\mathrm{c}}} = \frac{|{}^{d\gamma}/_{dT}|}{\mu L k}\frac{\dot{Q}}{v_{\mathrm{scan}}} \quad (24)$$

We fixed process speed, $v_{scan} = 200$ mm s^{-1} ($\tau_{\mathrm{l}} = 50$ μs), the list of S for AgNP shown in Supplementary Table 2.

Supplementary Note 5. Boundary condition between AgNP layer and substrate in heat transfer analysis

In derivation of characteristic temperature difference, ΔT, a heat flux across interface between AgNP and PET was treated as zero, i.e. adiabatic condition. This assumption can be verified by two unique natures of the process; 1) The processing time of laser melting is very short (50 μs) and 2) There is distinctive thermal diffusivity difference between AgNP layer and PET substrate. Within these conditions the heat provided by laser should not propagate through to the substrate. Comparing the propagated thermal lengths of each parts during 50 μs, the length for AgNP is about 26 times longer than the PET substrate (thermal length of the AgNP : 61.1 μm, thermal length of the PET substrate : 2.33 μm).

Furthermore, the time dependent FEM simulation (conditions are listed in Supplementary Table 5) was implemented for clear verification. As depicted in Supplementary Figure 3, simulated heat flow rate across the interface between AgNP and PET is negligibly small compared to the heat applied by laser irradiation, indicating that the majority heat exists in the high conductive region. Meanwhile, if the substrate has a high thermal conductivity, e.g. copper, thermal lengths of each materials should have the same order of magnitude, expecting that the heat flow propagates to both substrate and AgNP layer. We implemented the simulation replacing the substrate into Copper. The heat flow rate across interface was higher compared to the previous one, showing that 90 % of the irradiated power was propagated to the substrate (Supplementary Figure 3b).

Through above consideration, the boundary condition for the surface between AgNP layer and PET substrate can be approximated by adiabatic (i.e. insulated), and the heat transfer would be dominated by the AgNP layer.

Supplementary Note 6. Additional discussion on the laser profile and shape

As shown in Supplementary Figure 4a, relatively smooth laser power distribution (photon energy distribution) can be simply classified into three categories: 1) Power density focused at the spot center, 2) Power density spread uniformly, and 3) Beam has the maximum power density at the edge. Since irradiated laser power must be same in each cases, the former case has smaller beam size with high effective power density; thus, creating a sharper temperature gradient (Large amount of heat flux focused at the center with insufficient time for heat propagation). Therefore, effective power density will be the main parameter which determines the temperature gradient.

For further investigation of the effect of the laser beam shape, as illustrated in Supplementary Figure 4b, we compared the distinctively different cases whether the shape is vertically or horizontally aligned to the laser scanning direction. The shape will determine the time of irradiation in a certain area as $\tau_{\mathrm{l}} = l_{\mathrm{i}}/v$. The relation of the S number can be converted in to functions containing process parameters such as heat flux and laser time.

$$\mathrm{S} = \frac{\left|{}^{d\gamma}/_{dT}\right|}{\mu L k}\frac{\dot{Q}}{v_{\mathrm{scan}}} = \frac{\left|{}^{d\gamma}/_{dT}\right|}{\mu L k}\frac{q'' L^2}{L/\tau_{\mathrm{l}}} = \frac{\left|{}^{d\gamma}/_{dT}\right|}{\mu k} q''\tau_{\mathrm{l}} \tag{25}$$

As the formula, a laser distribution with a high energy density or a beam shape aligned along the scanning direction will tend to form irregular balling with high S number. To illustrate, reducing the beam diameter of the laser spot with a constant power will increase the effective power density. As a result, the temperature difference, which is the driving parameter of Marangoni convection, will be increased and the characteristic time of the flow will be reduced. If the beam shape is longer in the scanning direction while maintaining the power density, the temperature difference will not change, but the laser irradiation time itself becomes longer and the time for the AgNP layer in liquid phase will be increased. Both of these cases will have a high S number and will form an irregular balling structure. To obtain the unique structure (self-generated regular wavy structure) in our work, an appropriate power density and beam shape should be determined from the aforementioned argument.

Supplementary Note 7. The relation between contacted area and applied pressure

The contact problem of elastic body with rigid arbitral surface was investigated for model the situation of operating the sensor. In condition that $\frac{\delta}{\lambda} \ll 1$, shear strain of upper layer is negligible so that longitudinal strain should be dealt dominantly. Relations between the two-dimensional average pressure, $p(x)$ and normal displacement, $u_{\mathrm{z}}(x)$ can be derived as followed[4]:

$$\int_{-a}^{a} \frac{p(s)}{x-s} ds = -\frac{\pi E}{2(1-\nu^2)} \frac{du_{\mathrm{z}}}{dx} \tag{26}$$

If we let the right hand side be $\mathrm{g}(x)$, made up from a combination of material parameters and displacement gradient, the equation has a general solution of the form[5] as

$$p(x) = \frac{1}{\pi^2\sqrt{a^2-x^2}} \int_{-a}^{a} \frac{\sqrt{a^2-s^2}g(s)}{x-s} ds + \frac{C}{\pi^2\sqrt{a^2-x^2}} \tag{27}$$

where C is integral constant which denotes the average pressure among the contact region ($\mathrm{C} = \pi \int_{-a}^{a} F(s)ds$). If we take The equation above can be solved in which that u_{z} is of polynomial form : $u_{\mathrm{z}}' = -A_{\mathrm{n}}\mathrm{x}^{\mathrm{n}}$. Sinusoidal displacement, $u_{\mathrm{z}} = \delta\cos(\frac{2\pi}{\lambda}x)$, can be expressed as polynomial form by Taylor series. The pressure distribution for single term of polynomial nth power ($u_{\mathrm{z}}' = -A_{\mathrm{n}}x^n$) is shown as

$$p_{\mathrm{n}}(\mathrm{x}) = -\frac{EA_{\mathrm{n}}a^{n+1}}{2(1-\nu^2)\pi} \frac{I_{\mathrm{n}}}{\sqrt{a^2-x^2}} + \frac{P_{\mathrm{n}}}{\pi\sqrt{a^2-x^2}} \tag{28}$$

$$I_{\mathrm{n}} = \pi\left\{\left(\frac{x}{a}\right)^{n+1} - \frac{1}{2}\left(\frac{x}{a}\right)^{n-1} - \frac{1}{8}\left(\frac{x}{a}\right)^{n-3} - \cdots - \frac{1\cdot3\cdot5\cdots(n-3)}{2\cdot4\cdots n}\frac{x}{a}\right\}$$

for even n,

$$= \pi\left\{\left(\frac{x}{a}\right)^{n+1} - \frac{1}{2}\left(\frac{x}{a}\right)^{n-1} - \frac{1}{8}\left(\frac{x}{a}\right)^{n-3} - \cdots - \frac{1\cdot3\cdot5\cdots(n-2)}{2\cdot4\cdots(n+1)}\right\} \tag{29}$$

for odd n,

Using the continuity boundary conditions ($p(a) = p(-a) = 0$) and conditions for avoiding the

singularity of the equation, the relation between the average external pressure (P_{n}) for single term of polynomial and projected contact length (a) can be derived as

$$P_{\mathrm{n}} = \frac{Ea^{n+1}}{2(1-\nu^2)} A_{\mathrm{n}} I_{\mathrm{n}}(a) \tag{30}$$

we set the coefiicient A_{n} be counterpart of expanded cosine function,

$$A_{\mathrm{2n-1}} = \frac{2\pi\delta}{\lambda}\left(\frac{2\pi}{\lambda}\right)^{2n-1} \frac{(-1)^{n-1}}{(2n-1)!}$$
$$A_{\mathrm{2n}} = 0 \tag{31}$$

The final expression of nth approximated external pressurue should contain from 1 to n terms.

$$P = \sum_{n=1}^{\infty} P_{\mathrm{n}} = \sum_{n=1}^{\infty}\left[\frac{Ea^{2n}}{(1-\nu^2)} \frac{\pi\delta}{\lambda}\left(\frac{2\pi}{\lambda}\right)^{2n-1} \frac{(-1)^{n-1}}{(2n-1)!} I_{\mathrm{2n-1}}(a)\right] \tag{32}$$

This relation can be expressed identical to equation 1 in manuscript.

$$P[\mathrm{N\ m^{-1}}] = \frac{\pi^2 E\delta}{\lambda(1-\nu^2)}\left[\frac{1}{1!}\left(\frac{2\pi}{\lambda}\right)a^2 J_1 - \frac{1}{3!}\left(\frac{2\pi}{\lambda}\right)^3 a^4 J_3 + \frac{1}{5!}\left(\frac{2\pi}{\lambda}\right)^5 a^6 J_5 - \cdots\right] \tag{33}$$

where $J_{\mathrm{n}} = 1 - \frac{1}{2} - - \ \frac{1}{8} - \cdots - \ \frac{1\cdot3\cdot5\cdots(n-2)}{2\cdot4\cdots(n+1)}$

To construct the relation between external pressure and conductance, one should consider converting the projected area (a) to actual contact length (a_t). The relation between actual contact length (a_t) and projected length of contact area (a) can be given by

$$a_t = \frac{\lambda}{\pi} E(\frac{2\pi}{\lambda}a| - \left(\frac{2\pi\delta}{\lambda}\right)^2) \tag{34}$$

where $\mathrm{E}(\mathrm{x}|\mathrm{k}^2)$ is the elliptic integral of the second kind with parameter k^2 (Figure 3c(B-1)). Actual contact length directly affects the bridging current between the electrodes as depicted in Figure 3d of manuscript.

Supplementary Note 8. Parallel micro resistance approximation

The micro resistors appeared by the external pressure are arranged along interdigitated electrodes periodically (Supplementary Figure 6a). When the interval between cells is larger than contact area, $a_{\mathrm{t}} \ll \lambda$, electrical current started at one side of micro resistance flows heading to its facing counterpart (Supplementary Figure 6b). So that some cell of micro resistor cannot influence electrically to another cell. Therefore, the total impedance of one pair of interdigitated electrode can be approximated as an impedance of parallel connection of single cells. The charge conservation equation was solved by a simple FEM to confirm the independence between each cells (Supplementary Figure 6c, d). The horizontal current density was visualized to show distinct electrical separation of each cells. In a typical sensor working range, the error between total resistance and parallel approximation was only about 4.34%. (Supplementary Figure 6c)

Supplementary Note 9. Calculation of single cell micro resistance

Unlike the usual rectangular resistor where the equipotential line is parallel to the electrodes, the micro resistors between interdigitated electrodes have nonlinear equipotential contour (Supplementary Figure 6b). The geometrical resistance can be obtained analytically using the Schwarz-Cristoffel mapping to calculate the resistance of such resistors[12]. Specify the position of vertexes defining geometry in the complex plane. The vertexes of micro resistor cell as P, Q, R, and S. resistor's electrode position is identified by A, B, C, and D (Supplementary Figure 7b). Through three conformal mapping steps (Supplementary Figure 7), it is possible to convert the physical plane, *Z*-plane to imaginary one, χ-plane whose equipotential line is parallel to each other. With property of conformal transformation, conservation of local angle preserves the geometrical resistance. First step of transformation is that *Z*-plane to *t*-plane by following manner (Supplementary Figure 7a, b).

$$\mathrm{t} = \mathrm{sn}^2(mz, k) \tag{35}$$

where $\mathrm{u} = \mathrm{sn}^{-1}x = \int_0^x \frac{dt}{\sqrt{(1-t^2)(1-k^2t^2)}}$, Jacobi elliptic function, $\mathrm{m} = \frac{K}{l} = \frac{K'}{l'}$ k is chosen by $\frac{\mathrm{K}'}{\mathrm{K}} = \frac{l'}{l}$ relation,

$$K = \int_0^1 \frac{dt}{\sqrt{(1-t^2)(1-k^2t^2)}}, K' = \int_0^1 \frac{dt}{\sqrt{(1-t^2)(1-k'^2t^2)}}, \quad k' = \sqrt{1-k^2} \tag{36}$$

Secondly, for matching the vertexes of micro resistor and its electrode, the transformation

$$\mathrm{u} = \frac{d-b}{b-a}\frac{t-a}{d-t} \tag{37}$$

was applied (Supplementary Figure 7c). Finally, inverse transformation of first step,

$$\mathrm{u} = \mathrm{sn}^2(\chi, \lambda), \frac{1}{\lambda^2} = \frac{d-b}{b-a}\frac{c-a}{d-t} \tag{38}$$

leads *t*-plane to χ-plane configuration, which easily calculates the geometrical resistance provided by the ratio of sides of rectangle $\frac{F}{F'}$ (Supplementary Figure 7d). If we non-dimensionalize the lengths as $X = l/l'$, $Y = a_{\mathrm{t}}/l'$, an excellent approximation of geometrical resistance was investigated with conditions $X << 1, Y < 0.5$.[6] The sensor's geometry and working circumstances satisfy above

condition, we safely approximated the geometrical resistance as

$$G \cong K'(k)/K(k), \text{ where } k \cong \tanh[(\frac{\pi}{2}\left(\frac{Y}{X}\right)]. \quad (39)$$

Supplementary Note 10. Influence of AgNW quantity on sensor performace

As the nanowire is randomly distributed over the polymer, the NW composite could be treated as a continuous metal layer. Since the output signal of the sensor is related with the micro-scale contact between the NW composite and the corrugated electrode, the sensitivity and the electrical response will be maintained with the altered nanowire density. As shown in Supplementary Figure 8a, the nanowire density was controlled in three different conductivity. Supplementary Figure 8b depicts that the output signals are identical to the theoretical value, which proves that the concentration is irrelevant to the electrical response. Yet, the low density of nanowires near the percolation threshold may have issues in stability and repeatability in signals.

Supplementary Note 11. Absorbance of AgNP layer on 532 nm laser beam

The AgNP layer aborbes the heat flux of irradiated laser power in consideration of absorbance.

$$q'' = aq''_{\mathrm{laser}} \quad (40)$$

We measured the absorbance *a* of AgNP layer near 532 nm, wavelength of irradiated laser beam (Sprout-G-5W, Lighthouse Photonics, U.S.). The spectrophotometer (Cary 5000, Cary) measured the reflectance and transmittance of AgNP layer as shown Supplementary Figure 5. The absorbance can be calculated by reflectance, 0.47 as dictated in Supplementary Table 3 for 532 nm.

Supplementary Note 12. Detailed information of laser sintered electrodes

Detailed information regarding laser sintering process is shown in Supplementary Figure 9. Supplementary Figure 9a depicts the TEM image of the nanoparticle ink with average diameter of 40 nm. Nanoparticle ink is spin-coated on PET in 200 rpm for 60 sec, and uniformly distributed nanoparticle layer is prepared as shown Supplementary Figure 9b. The thickness of the spin-coated nanoparticle layer is around 1 μm as the SEM image is Supplementary Figure 9c. The magnified SEM image of the coated particles are shown in Supplementary Figure 9d. The optical system for laser sintering fabrication is schematically shown in Supplementary Figure 9e.

Flexibility of the electrode is shown in Supplementary Figure 14a, b. Ag microstructure is bent in 3mm curvature and the durability of the structure is shown in Supplementary Figure 14b (5000 cycles in 1 Hz condition). The surface morphology of the Ag structure is captured in a larger view by the 3D surface profiler in order to investigate the uniformity of the structure (Supplementary Figure 14c). Furthermore, the conductivity of the Ag microstructure is measured by the 4-point probe method. By the general relationship, $\rho/t = \frac{V}{I}\frac{\pi}{ln2}$, the conductance of the structure can be calculated by the measured resistance (V/I=0.0956 Ω) and the thickness of the Ag structure (t = 1 μm). The resultant conductance of the structure is therefore calculated by 2.32×10^{6} S m^{-1}.

Supplementary Note 13. Reliability and response time of pressure sensor

Various experiments to evaluate the response time and reliability of the sensor were conducted as shown in Supplementary Figure 10. The dynamic cycle response in 2Hz frequency is shown in Supplementary Figure 10a, b. Varying pressures in 300Pa, 1kPa, and 30kPa is applied for 10,000 cycles per each. The magnified cycle plot is depicted in Supplementary Figure 10b. To further explore the sensor's response time, calculated the time constant from the data was shown in Supplementary Figure 10c, d with the value of 24 ms.

Supplementary Note 14. 6-wired 3D touch system

We demonstrated the transparent and flexible pressure sensor, a single device which composed AgNW upper layer and laser sintered comb-drive Ag electrode lower layer. The upper part of pressure sensor has excellent and uniform electrical conductivity of AgNW percolation network. If bias applies to two facing side of upper layer, electrical potential uniformly drops along perpendicular line to biased side. In this situation, voltage read by a probe contacted some point would represent the position information, distance between biased electrode and a probe. For another two facing side, same procedure can be conducted providing distance information for perpendicular axis of first one. Let first biased electrodes be x-axis, consecutive counterpart be y-axis. These voltage values set can be interpreted the 2D information of probe on upper layer. We treated the one side of comb-drive electrode of lower layer as voltage probe, connecting additional four wire to each four side of upper layer. Overall connected wires needed 6 wire, additional 4 wire to original pressure sensor. Since data reading pin is connected only to one side of comb-drive electrode, 3 measure processes are conducted sequentially. Conducting 3 measure steps, pins related other procedures should be electrically isolated avoiding interference between each measuring data (Supplementary Table 4). The 3D information measuring scheme is following as Supplementary Figure 11. Furthermore, by applying additional resistors and voltage measuring units[13], the system also has potential for multi-touch measurement.

Supplementary Note 15. Multi-pixel array system

Single device of transparent and flexible pressure sensor can be easily integrated compactly by advantages of laser direct writing. We confirmed in manuscript (Figure 4e-k) that integrated system containing 100 small scale ($5\text{mm} \times 5\text{mm}$ for each pixel) pressure sensors worked with outstanding performance same as original scale device. For operating the system, 10 row and 10 column, 20 data lines should be connected to each pixels. The microcontroller (Arduino Mega 2560) with FPC electronics are combined to measuring pressure data of each pixel sequentially.

Supplementary Tables

τ_m	τ_l	τ_s
0.117 ns	50 μs	2.76 μs

Supplementary Table 1. Calculated time constants

$\dot{Q}$(mW)	S (Surface shaping number)
10	0.097
50	0.48
100	0.97
120	1.16
140	1.36

Supplementary Table 2. Various S values in processing laser powers

Property (symbol)	Value	reference
Surface tension gradient ($^{d\gamma}/_{dT}$)	$0.1869\ \mathrm{mN\ m^{-1}\ K^{-1}}$	[7]
Density (ρ)	$8600\ \mathrm{kg\ m^{-3}}$	[8]
Specific heat (c_p)	$0.28\ \mathrm{kJ\ kg^{-1}\ K^{-1}}$	[9]
Thermal conductivity (k)	$180\ \mathrm{W\ m^{-1}\ K^{-1}}$	[10]
Viscosity (μ)	$25.12\ \mathrm{mPa\cdot s}$	[11]
Absorbance (a)	0.47	Supplementary Figure 5

Supplementary Table 3. Thermofluidic properties of AgNP layer

	x-measure	y-measure	p-measure
x1	LOW	OPEN	OPEN
x2	HIGH	OPEN	OPEN
y1	OPEN	LOW	OPEN
y2	OPEN	HIGH	OPEN
p1	OPEN	Open	HIGH
p2	OPEN_measure	OPEN_measure	LOW_measure

Supplementary Table 4. Pins' state for each 3 measuring modes

Parameters	Value
Beam profile	Gaussian
Beam diameter (std. div. of Gaussian distribution)	5 µm
Beam power	90mW
Beam speed	200mm s^{-1}
Cell geometry	200 µm (x), 200 µm (y), 26 µm (z, 1 µm for AgNP, 25 µm for substrate)
Boundary conditions for faces	Heat sink to ambient (except upper face), Natural convection (upper face)
AgNP properties	Supplementary Table 3
PET properties	Values in library of COMSOL Multi Physics
Copper properties	Values in library of COMSOL Multi Physics

Supplementary Table 5. Conditions implementing the time dependent FEM simulation

Supplementary References

1. F. Incropera, D. Dewitt, T. Bergman, A. Lavine, *Foundation of heat transfer*, 6ed, Wiley, 2012.
2. Bäuerle, D. *Laser processing and chemistry*. (Springer, 2011).
3. Johnson, P. B. & Christy, R.-W. Optical constants of the noble metals. *Phys. Rev. B* 6, 4370 (1972).
4. Johnson, K. L. & Johnson, K. L. *Contact mechanics*. (Cambridge university press, 1987).
5. Mikhlin, S. G. e. Singular integral equations. *Uspekhi Mat. Nauk* 3, 29-112 (1948).
6. Hall, P. M. Resistance calculations for thin film rectangles. *Thin Solid Films* 300, 256-264 (1997).
7. Moser, Z., Gasior, W. & Pstruś, J. Surface tension of liquid Ag-Sn alloys: experiment versus modeling. *J. of Phase Equilib.* 22, 254 (2001).
8. Kirshenbaum, A., Cahill, J. & Grosse, A. The density of liquid silver from its melting point to its normal boiling point 2450 K. *J. of Inorg. and Nucl. Chem.* 24, 333-336 (1962).
9. Engineering ToolBox, T. E. (2014). Metals - as Liquids. [online] Available at: https://www.engineeringtoolbox.com/liquid-metal-boiling-points-specific-heat-d_1893.html [Accessed Day 11. 2018].
10. Giordanengo, B., Benazzi, N., Vinckel, J., Gasser, J. & Roubi, L. Thermal conductivity of liquid metals and metallic alloys. *J of Non-cryst. Solids* 250, 377-383 (1999).
11. Strauss, S. W. The temperature dependence of the viscosity of liquid metals. *Nucl. Sci. and Eng.* 12, 436-439 (1962).
12. Bowman, F. *Introduction to elliptic functions: with applications*. English Universities Press London, (1953).
13. Calpe, J., Medina, I., Carbajo, A. & Martínez, M. J. AD7879 Controller Enables Gesture Recognition on Resistive Touch Screens. *Analog Dialogue* 45-06 (2011)
