# Supplementary material for: Transparent wearable three-dimensional touch by self-generated multiscale structure
Source: Nat Commun. 2019 Jun 13;10:2582. doi: 10.1038/s41467-019-10736-6 (PMC6565712; doi:10.1038/s41467-019-10736-6)
Supplement: Supplementary file 2 — Description of Additional Supplementary Files [file 41467_2019_10736_MOESM2_ESM.pdf]

Description of Additional Supplementary Files

Supplementary Movie 1.

Drawing G-clef with transparent 3D touch

Supplementary Movie 2.

Drawing 3D structured Spring with 3D touch

Supplementary Movie 3.

Implementation with wireless module
